# The risk of adverse clinical outcomes following treatment of Plasmodium vivax malaria with and without primaquine in Papua, Indonesia

**DOI:** 10.1371/journal.pntd.0008838

**Published:** 2020-11-11

**Authors:** Kamala Thriemer, Jeanne-Rini Poespoprodjo, Enny Kenangalem, Nicholas M. Douglas, Paulus Sugiarto, Nicholas M. Anstey, Julie Anne Simpson, Ric N. Price

**Affiliations:** 1 Global Health Division, Menzies School of Health Research and Charles Darwin University, Darwin, Australia; 2 Centre for Child Health and Department of Child Health, Faculty of Medicine, Public Health and Nursing, Universitas Gadjah Mada, Yogyakarta, Indonesia; 3 Timika Malaria Research Programme, Papuan Health and Community Development Foundation, Timika, Papua, Indonesia; 4 Mimika District Hospital, Timika, Papua, Indonesia; 5 Division of Medicine, Royal Darwin Hospital, Darwin, Australia; 6 Mitra Masyarakat Hospital, Timika, Indonesia; 7 Centre for Epidemiology and Biostatistics, Melbourne School of Population and Global Health, University of Melbourne, Melbourne, Australia; 8 Centre for Tropical Medicine, Nuffield Department of Clinical Medicine, University of Oxford, Oxford, United Kingdom; 9 Mahidol-Oxford Tropical Medicine Research Unit (MORU), Faculty of Tropical Medicine, Mahidol University, Bangkok, Thailand; Vienna, AUSTRIA

## Abstract

The widespread use of primaquine (PQ) radical cure for *P*. *vivax*, is constrained by concerns over its safety. We used routinely collected patient data to compare the overall morbidity and mortality in patients treated with and without PQ without prior testing of Glucose-6-Phosphate-Dehydrogenase (G6PD) deficiency in Papua, Indonesia, where there is a low prevalence of G6PD deficiency. Records were collated from patients older than 1 year, with *P*. vivax infection, who were treated with an artemisinin combination therapy (ACT). The risks of re-presentation, hospitalization, major fall in haemoglobin and death within 30 days were quantified and compared between patients treated with and without PQ using a Cox regression model. In total 26,216 patients with *P*. *vivax* malaria presented to the hospital with malaria during the study period. Overall 27.56% (95% Confidence Interval (95%CI): 26.96–28.16) of 21,344 patients treated with PQ re-presented with any illness within 30 days and 1.69% (1.51–1.88) required admission to hospital. The corresponding risks were higher in the 4,872 patients not treated with PQ; Adjusted Hazard Ratio (AHR) = 0.84 (0.79–0.91; p<0.001) and 0.54 (0.41–0.70; p<0.001) respectively. By day 30, 14.15% (12.45–16.05) of patients who had received PQ had a fall in haemoglobin (Hb) below 7g/dl compared to 20.43% (16.67–24.89) of patients treated without PQ; AHR = 0.66 (0.45–0.97; p = 0.033). A total of 75 (0.3%) patients died within 30 days of treatment with a mortality risk of 0.27% (0.21–0.35) in patients treated with PQ, compared to 0.38% (0.24–0.60) without PQ; AHR = 0.79 (0.43–1.45; p = 0.448). In Papua, Indonesia routine administration of PQ radical cure without prior G6PD testing, was associated with lower risk of all cause hospitalization and other serious adverse clinical outcomes. In areas where G6PD testing is not available or cannot be delivered reliably, the risks of drug induced haemolysis should be balanced against the potential benefits of reducing recurrent *P*. *vivax* malaria and its associated morbidity and mortality.

## Author summary

The radical cure of vivax malaria requires killing the blood stages and the dormant liver stages (hypnozoites) of *Plasmodium vivax*. Primaquine (PQ) is currently the only widely available drug to kill hypnozoites, but its use is hampered by concerns over haemolysis in patients with Glucose-6-Dehydrogenase (G6PD) deficiency. At a recent round table discussion of the Asia Pacific Malaria Elimination Network (APMEN) policy makers prioritised further studies to determine the risk-benefit of PQ in different endemic settings.

We used a large dataset of more than 26,000 patient treated in a routine care setting in Papua, Indonesia over a 9-year period to explore the risk of adverse outcomes following PQ. Even though patients were treated with PQ without G6PD testing, those patients receiving PQ had a 20% lower risk of all cause re-presentation and 50% lower risk of hospitalization than those not treated with PQ. The risk of mortality was 20% lower, although this didn’t reach statistical significance.

Great emphasis is placed on the risks of PQ induced haemolysis, inhibiting its widespread use. It is critical that these risks are interpreted in the context of the substantial benefits of reducing the recurrence of episodes of malaria and associated morbidity and mortality. Our analysis provides reassuring evidence that, in an area with a high burden of malaria and low G6PD prevalence the beneficial effects of PQ outweigh its risks.

## Introduction

In the last decade major gains have been made in malaria control, but successes have been less apparent for *P*. *vivax* than for *P*. *falciparum*. Unlike *P*. *falciparum*, *P*. *vivax* forms dormant liver stages (hypnozoites) that can reactivate weeks to months after an initial infection, causing recurrent parasitaemia (relapses). Whereas schizontocidal drugs are effective in killing blood stage parasites and reducing acute symptoms, they have no efficacy against the dormant liver stages of the parasite. The parasite’s propensity to recur weeks or months after initial infection results in recurrent febrile illness and a cumulative risk of anaemia and associated morbidity and mortality [1–3]. Relapsing infections are also a major contributor to the ongoing transmission, that must be addressed if targets for malaria elimination are to be achieved [4].

The only widely available drug to kill hypnozoites and therefore prevent relapsing infection is primaquine (PQ), which is administered with schizontocidal treatment in a combination known as radical cure. The effectiveness of PQ is limited by the prolonged treatment course (14 days) recommended by most national malaria control programs, and concerns over its safety, particularly the risk of acute haemolysis in individuals with glucose-6-phosphate-dehydrogenase (G6PD) deficiency [5]. G6PD deficiency is one of the most common enzymopathies worldwide, present in up to 30% of the population in some malaria endemic regions [6]. The current World Health Organisation (WHO) antimalarial treatment guidelines recommend that all patients should be tested for G6PD deficiency prior to administering PQ [7], however in reality testing is often not feasible [5]. If G6PD testing is not available, then the WHO guidelines recommend that a local risk-benefit assessment should guide antimalarial policy [7]. The risk-benefit considerations for PQ deployment depend on a variety of host, parasite and environmental factors, including the risk and frequency of relapse, access to healthcare, and the prevalence and local variants of G6PD deficiency.

We undertook a retrospective cohort study of patients with *P*. *vivax* malaria in Papua, Indonesia, an area endemic for *P*. *vivax* strains with a short relapse periodicity and low G6PD prevalence [8]. The aim of the study was to quantify the risks of all-cause morbidity and mortality following treatment with or without PQ in a routine health care setting without prior testing for G6PD.

## Methods

### Ethics and data access

The study is a retrospective analysis that was conducted on routine data collected from a healthcare facility in Papua, Indonesia. Although individual consent was not collected from patients, all data were anonymised. Ethical approval was obtained from the Human Research Ethics Committee of the Northern Territory (HREC 10.1397) and the Health Research Ethics Committees of the University of Gadjah Mada (KE/FK/544/EC), Indonesia.

### Study design

The study was designed as a retrospective cohort study of routinely collected patient data to compare the morbidity and mortality of patients treated with and without PQ.

### Study site

The climate, geography, malaria endemicity and demographics of the study site have been described previously [8–10]. In brief, the study area lies in south-central Papua, Indonesia, and has perennial malaria transmission with approximately half of the malaria infections due to *P*. *vivax* [8].

The study was undertaken in patients presenting to the Rumah Sakit Mitra Masyarakat (RSMM) hospital, in Timika, Papua province, Indonesia, between April 2004 and December 2013. RSMM is the largest health care facility in Timika and one of two public hospitals in the district. Since March 2006 local guidelines have recommended dihydroartemisinin-piperaquine (DHP) as the first line treatment for uncomplicated malaria due to any *Plasmodium* species [9]. PQ radical cure is recommended for non-pregnant women and children over the age of 1 year presenting with *P*. *vivax* malaria. Before March 2006 the recommended total dose was 3.5mg/kg administered over 14 days. In March 2006 local guidelines were changed to recommend a total dose of 7mg/kg administered over 14 days. For patients presenting to the hospital outpatient clinic, PQ was commenced at the same time as schizontocidal treatment, whereas in inpatients, PQ was administered once clinical recovery had begun and patients were able to take oral medication.

In Indonesia, treatment guidelines do not recommend routine testing for G6PD deficiency to guide the administration of PQ. In a cross-sectional survey in 2013, the prevalence of severe or intermediate G6PD deficiency was 2.6% and was slightly lower in individuals of highland Papuan ethnicity (2.1%) compared to lowlanders and non-Papuans (2.6% and 2.8%) respectively [8].

### Laboratory and data collection procedures

Data from all patients presenting to the RSMM between 2004 and 2013 were recorded in a Q-Pro database by hospital administrators. The following data were recorded: the patients’ unique identification number, demographic details, date of visit and duration of hospital admission, clinical diagnosis and laboratory investigations. Details on prescriptions at the hospital pharmacy were recorded in a separate database and linked to the same unique hospital record number (HRN).

Hospital protocols require that all patients attending the outpatient clinic with fever or symptoms consistent with malaria and all inpatients regardless of their clinical presentation, were tested for malaria by microscopic examination of Giemsa-stained blood films. In some instances, HRP2 based rapid diagnostic tests were used for additional confirmation of *falciparum* malaria.

### Data preparation

The HRN was used to link clinical and laboratory data with the pharmacy records. Since recurrent episodes of *P*. *vivax* malaria are associated with a cumulative risk of anaemia and both direct and indirect mortality [11,12], the analysis was limited to patients presenting with their first episode of *P*. *vivax*, alone or mixed with another *Plasmodium* species, and all associated clinical events occurring within the ensuing 30 days. Pharmacy records reported the number of tablets prescribed and the actual mg per kg dose of PQ prescribed were derived for each individual patient by applying estimated mean body weights within each age, sex, and ethnicity strata derived from a cross sectional survey [13]. The total dose of PQ administered was categorised as described previously [14]: (i) No PQ (matching antimalarial prescription data, but no PQ prescribed), (ii) single dose PQ (>0mg/kg and <1.5mg/kg total dose), (iii) low dose PQ (≥1.5mg/kg and <5mg/kg total dose), (iv) high dose PQ (≥5mg/kg total dose), (v) unknown dose PQ (matched PQ prescription record, but unable to determine dose in mg per kg).

### Study population

**Fig 1:**
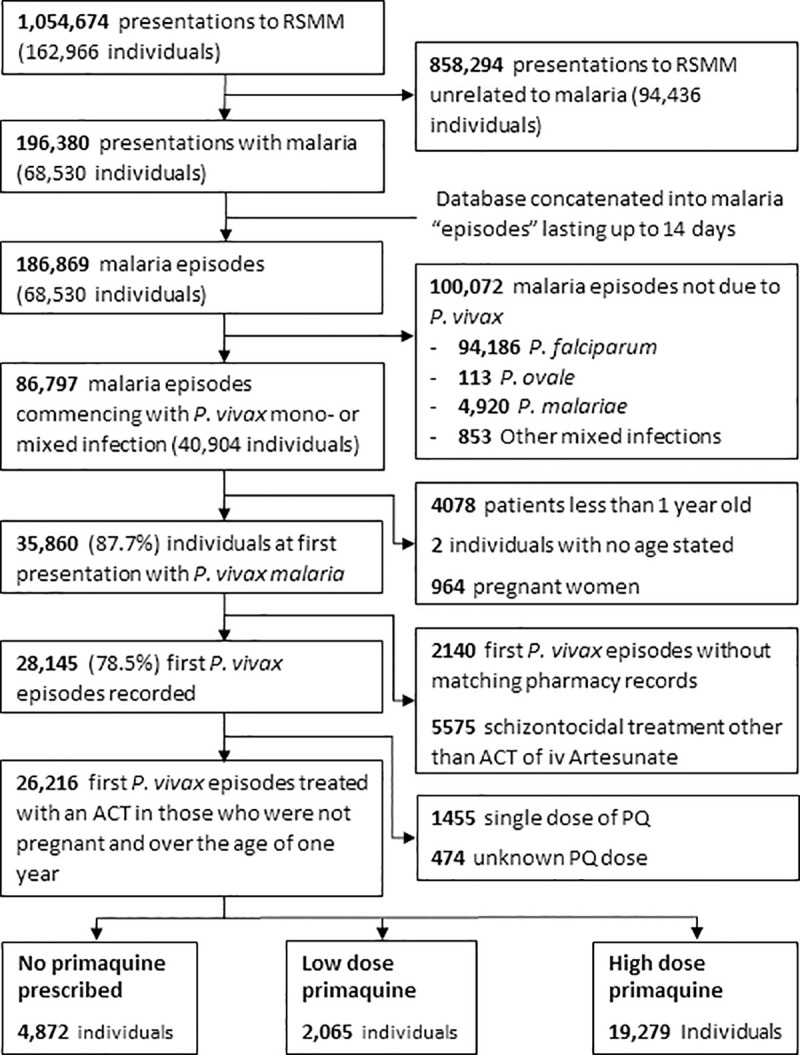
Study Profile.

Patients with microscopically confirmed *P*. *vivax* parasitaemia, either mono-infection or part of a mixed infection, were included in the analyses if they were treated with an artemisinin combination therapy (ACT). Patients treated with non-ACT regimens prior to policy change in 2006 were excluded, since chloroquine resistant *P*. *vivax* is highly prevalent in Papua and its poor efficacy is associated with a high risk of adverse events attributable to parasitological failure [15]. In this location ACTs are highly effective against the blood stages of *P*. *vivax* [16,17]. PQ is not recommended in patients less than one year of age or in pregnant women and so these patients were excluded from the analysis. Patient were also excluded if they were prescribed a single dose of PQ or the dose was unknown (Fig 1).

### Statistical analyses

The analysis was conducted according to an a priori statistical analysis plan (S1 Text) and is reported according to RECORD guidelines (S1 Table). Three endpoints were assessed, each occurring within 30 days of the initial presentation, due to all causes not just malaria: i) re-presentation to hospital, ii) hospital admission and iii) death. Further endpoints were limited to patients who had a haemoglobin (Hb) measurement of first presentation and again within 30 days and included a Hb fall below 7g/dl, below 5g/dl and a fractional fall >25% to an absolute Hb < 7g/dl. The primary comparison was between patients treated without PQ and patients treated with any dose of PQ (either low or high dose). Patients treated with high dose PQ were compared with those receiving no PQ, in the following pre-specified subgroups: (i) age category (1 to <5 years, 5 to <15 years, ≥15 years), (ii) 12 months pre and post the largest PQ stock outage in 2007, (iii) patients initially treated as outpatients and (iv) patients of lowland and non-Papuan ethnicity.

The cumulative risks of each of the outcomes were estimated using Kaplan Meier survival curves. Patients with an event on day 1 or 2 were censored on that day under the assumption that this event was related to the initial vivax episode rather than the drug treatment. Hazard Ratios for the association between treatment with/without PQ and each of the outcomes were calculated using a Cox regression model. In view of significant changes in morbidity and mortality of malaria in this area [9], all multivariable models were stratified by year and adjusted for the following covariates: admission status, ethnicity, sex and age. The haematological outcomes were assessed using Kaplan Meier and Cox regression analyses with the models run separately for the total period (days 3–30) and the periods day 3–14 and day 15–30 to assess the risks during and after PQ treatment, respectively. The number needed to treat (NTT) to prevent one clinical endpoint were calculated as described previously [18].

## Results

### Baseline

Between April 2004 and December 2013, 35,860 non-pregnant patients more than 1 years old presented to the hospital for the first time with *P*. *vivax* malaria (alone or as part of a mixed *Plasmodium* infection). A total 2,140 patients were excluded because they had no matching pharmacy records and 5,575 were excluded because they received schizonticidal treatment other than ACT or injectable artesunate. A further 1,455 only received a single dose PQ and for 474 patients the PQ dose was missing (Fig 1).

Of the remaining 26,216 patients included in the analysis, 19,279 (73.54%) received a high dose of PQ, 2,065 (7.88%) were prescribed a low dose of PQ, and 4,872 (18.58%) received no PQ. The baseline characteristics of patients treated with or without PQ were similar (Table 1). The main difference between treatment arms was that inpatients were less likely to be treated with PQ (71.68%, 2000/2,790), compared to outpatients (82.57%, 19,344/23,426). Patients with anaemia (Hb<10g/dl) were also less likely to be treated with PQ (85.03%; 4,203/4,943) compared to those without anaemia (74.68%; 3003/4,021).

**Table 1:** Baseline demographics of the study population.

	No PQ		Low dose PQ		High dose PQ	
	N = 4,872		N = 2,065		N = 19,279	
	No.	%	No.	%	No.	%
**Initial Species**						
Pure *P*. *vivax*	3,658	75.1	1,458	70.6	13,245	68.7
Mixed *P*.*vivax/P*.*falciparum*	1,214	24.9	607	29.4	6,034	31.3
**Age**						
1 to <5years	1,161	23.8	149	7.2	3,887	20.2
5 to <15years	831	17.1	334	16.2	3,479	18.0
≥15years	2,880	59.1	1,582	76.6	11,913	61.8
**Sex**						
Female	2,484	51.0	1,170	56.7	8,823	45.8
Male	2,388	49.0	895	43.3	10,456	54.2
**Ethnic Group**						
Non-Papuan	847	17.4	349	16.9	2,915	15.1
Highland	3,627	74.5	1,558	75.6	14,683	76.3
Lowland	394	8.1	155	7.5	1,658	8.6
**Year**						
2005	37	0.8	30	1.5	2	0
2006	205	4.2	561	27.2	1,110	5.8
2007	2,278	46.6	189	9.2	905	4.7
2008	712	14.6	162	7.8	1,958	10.2
2009	646	13.3	175	8.5	2,406	12.5
2010	141	2.9	174	8.4	3,086	16.0
2011	119	2.4	180	8.7	3,294	17.1
2012	196	4.0	276	13.4	3,479	18.0
2013	538	11.0	318	15.4	3,039	15.8
**Initial admission status**						
Inpatients	790	16.2	323	15.6	1,677	8.7
Outpatient	4,083	83.8	1,742	84.4	17,602	91.3
**Initial anaemia (Haemoglobin<10g/dl) present**	N = 1,728		N = 811		N = 6,425	
Yes	740	42.8	417	51.4	3,786	58.9
No	988	57.2	394	48.6	2,639	41.1

Abbreviations: PQ = primaquine

Overall 24,148 (92.1%) patients were treated with DHA-Piperaquine and 1,720 (6.6%) with artesunate-amodiaquine. A total of 2,482 (9.6%) of patients were treated with intravenous artesunate alone or in combination.

### Risk of re-presentation to hospital within 30 days

**Fig 2:**
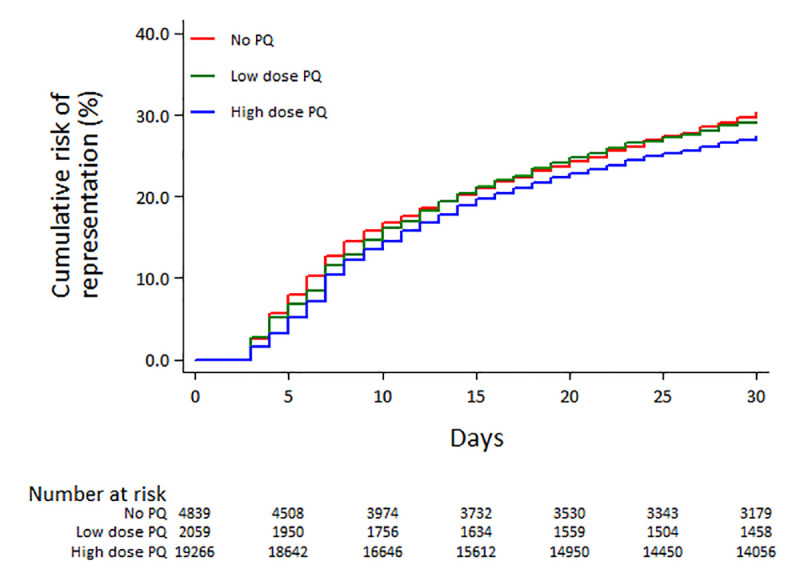
Risk of all cause re-presentation by primaquine (PQ) dose.

Overall 7,296 (27.83%) patients re-presented to hospital between 3 and 30 days after their initial presentation; 774 (10.61%) of these re-presentations were associated *P*. *vivax* (either alone or mixed infections), 457 (6.26%) with *falciparum* malaria, 17 (0.23%) with other malaria species and 6,048 (82.89%) were due to non-malarial illness. The cumulative risk of re-presentation at 30 days was 27.56% (95%CI: 26.96–28.16) for those with any PQ dose compared to 30.30% (95%CI: 29.01–31.64) in patients not treated with PQ (adjusted Hazard Ratio (AHR) = 0.84 (95%CI: 0.79–0.91); p<0.001) (Fig 2, Table 2). The number needed to treat (NNT) with PQ to prevent one re-presentation to hospital by day 30 was 24 (95%CI: 18–43).

**Table 2:** Risk of all cause re-presentation, hospitalization and death within 30 days after different doses of primaquine.

	PQ dose	N	Number of events	Cumulative risk in % (95%CI)	Unadjusted Hazard Ratio (95%CI)	p	Adjusted Hazard Ratio (95%CI)	p
**Re-presentation**	No PQ	4,872	1,426	30.30 (29.01–31.64)	Reference		Reference	
	Low Dose PQ	2,065	601	29.24 (27.32–31.26)	0.96 (0.87–1.06)	0.393	0.90 (0.81–1.00) ^1^	0.044
	High Dose PQ	19,279	5,269	27.38 (26.75–28.01)	0.88 (0.83–0.94)	<0.001	0.84 (0.78–0.90) ^1^	<0.001
	Any Dose PQ	21,344	5,870	27.56 (26.96–28.16)	0.89 (0.84–0.94)	<0.001	0.84 (0.79–0.91) ^1^	<0.001
**Hospitalization**	No PQ	4,872	107	2.71 (2.25–3.27)	Reference		Reference	
	Low Dose PQ	2,065	32	1.84 (1.30–2.59)	0.68 (0.46–1.00)	0.052	0.60 (0.39–0.92) ^2^	0.190
	High Dose PQ	19,279	294	1.67 (1.49–1.87)	0.62 (0.49–0.77)	<0.001	0.53 (0.40–0.69) ^2^	<0.001
	Any Dose PQ	21,344	326	1.69 (1.51–1.88)	0.62 (0.50–0.77)	<0.001	0.54 (0.41–0.70) ^2^	<0.001
**Death**	No PQ	4,872	18	0.38 (0.24–0.60)	Reference		Reference	
	Low Dose PQ	2,065	8	0.39 (0.19–0.78)	1.02 (0.44–2.35)	0.960	0.93 (0.38–2.28) ^3^	0.869
	High Dose PQ	19,279	49	0.25 (0.19–0.34)	0.67 (0.39–1.15)	0.114	0.77 (0.41–1.43) ^3^	0.403
	Any Dose PQ	21,344	57	0.27 (0.21–0.35)	0.70 (0.41–1.19)	0.191	0.79 (0.43–1.45) ^3^	0.448

Abbreviations: PQ = primaquine

^1^ Cox model stratified by year, and adjusted for sex, ethnicity, admission status and age. (full model presented in S1 Table)

^2^ Cox model stratified by year, and adjusted for sex, ethnicity and age. (full model presented in S2 Table)

^3^ Cox model stratified by year, and adjusted for sex, ethnicity, admission status and age. (full model presented in S3 Table)

When re-presentations were categorized as malaria or non-malaria related, patients treated with PQ had a low risk of re-presenting with malaria compared to those not treated with PQ (HR = 0.59 (95%CI: 0.52–0.67); p<0.0001), but there was no difference between treatment arms in the risk of representing with non-malarial illness (HR = 0.98 (95%CI: 0.92–1.05); p = 0.551). Females, children, those presenting with *P*. *vivax* mono-infection and those requiring hospital admission were significantly more at risk of re-presenting to hospital within 30 days (S2 Table). After controlling for these confounding factors, PQ was associated with a lower risk of re-presentation to hospital (AHR = 0.84 (95%CI: 0.79–0.91); p<0.001) compared to no PQ and this was apparent for both patients treated with low dose PQ (AHR = 0.90 (95%CI: 0.81–1.00); p = 0.044) and high dose PQ (AHR = 0.84 (95%CI: 0.78–0.90); p<0.001) (Table 2). After controlling for confounding factors, the type of ACT prescribed (DP or AAQ) was not correlated with the risk of re-presentation. In the *a priori* subgroup analyses, the risk of re-presentation in patients treated with a high dose PQ was also lower than that in patients not treated with PQ (Table 3).

**Table 3:** Subgroup analyses of high dose PQ versus no PQ for all-cause re-presentation, hospitalization and death within 30 days.

		Re-presentation		Hospitalization		Death	
	PQ dose	Adjusted Hazard Ratio (95%CI)	p	Adjusted Hazard Ratio (95%CI)	p	Adjusted Hazard Ratio (95%CI)	p
**Overall model**	No PQ	Reference		Reference		Reference	
	High dose PQ	0.84 (0.79–0.90)	<0.005	0.52 (0.39–0.68)	<0.005	0.77 (0.41–1.43)	0.403
**Age**^**1**^							
1 to <5 years	No PQ	Reference		Reference		Reference	
	High dose PQ	0.86 (0.76–0.98)	0.023	0.52 (0.35–0.77)	0.001	0.66 (0.19–2.27)	0.508
5 to <15 years	No PQ	Reference		Reference		Reference	
	High dose PQ	0.88 (0.73–1.07)	0.215	1.01 (0.37–2.78)	0.987	0.11 (0.006–1.80)	0.120
≥15 years	No PQ	Reference		Reference		Reference	
	High dose PQ	0.86 (0.79–0.95)	0.002	0.51 (0.35–0.76)	0.001	1.06 (0.48–2.34)	0.883
**2007 PQ stockout**^**2**^							
	No PQ	Reference		Reference		Reference	
	High dose PQ	0.91 (0.82–1.01)	0.075	0.74 (0.47–1.18)	0.209	1.56 (0.55–4.49)	0.405
**Admission status**^**3**^							
Outpatient	No PQ	Reference				Reference	
	High dose PQ	0.92 (0.77–0.90)	0.400	NA*		1.78 (0.47–6.70)	0.395
**Ethnicity**^**4**^							
Non Papuan	No PQ	Reference		Reference		Reference	
	High dose PQ	0.86 (0.70–1.05)	0.147	0.96 (0.29–3.18)	0.947	0.05 (0.006–0.43)	0.006
Lowland	No PQ	Reference		Reference		Reference	
	High dose PQ	0.77 (0.59–1.01)	0.062	0.30 (0.11–0.84)	0.021	3.45 (0.36–33.27)	0.284

Abbreviations: PQ = primaquine

^1^ Cox model stratified by year, and adjusted for sex, ethnicity and admission status.

^2^ Cox model stratified by year, and adjusted for sex, ethnicity, admission status and age.

^3^ Cox model stratified by year, and adjusted for sex, ethnicity and age.

^4^ Cox model stratified by year, and adjusted for age, sex and admission status.

* not applicable since this subgroup analysis was restricted to outpatients.

### Risk of hospitalization

**Fig 3:**
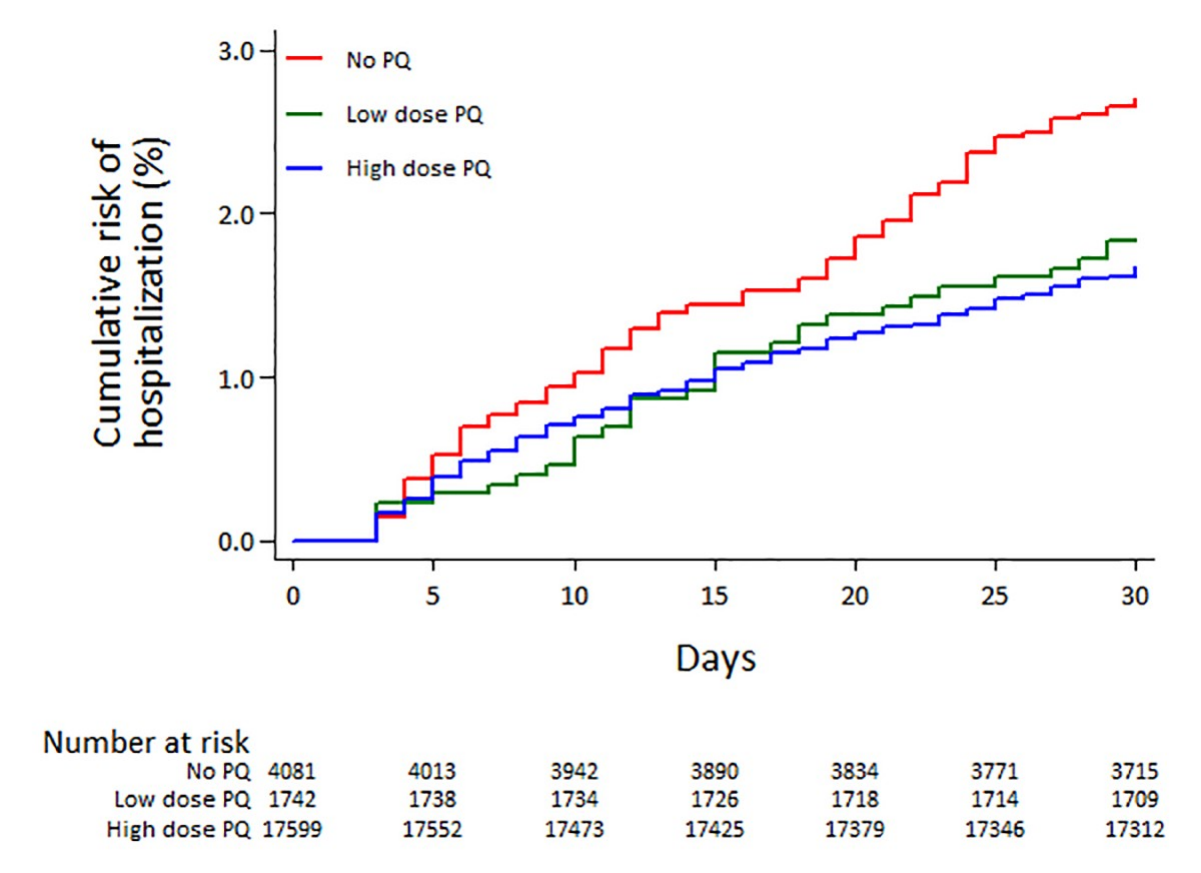
Risk of all cause hospitalization by primaquine (PQ) dose.

Overall 433 (1.85%) of 23,426 patients initially treated as outpatients, required admission to hospital between days 3 and 30. The cumulative risk of hospitalization at 30 days was 1.69% (95%CI: 1.51–1.88) in those treated with any dose of PQ compared to 2.71% (95%CI: 2.25–3.27) in patients not initially treated with PQ; HR = 0.62 (95%CI: 0.50–0.77), p<0.001 (Fig 3, Table 2). This remained significant after controlling for confounding factors (AHR = 0.54 (95%CI: 0.41–0.70); p<0.001) and when the dose of PQ was analysed separately (Table 2). Baseline risk factors for hospitalization are presented in S3 Table. The NNT with PQ to prevent one admission to hospital within 30 days was 81 (95%CI: 63–124). In the *a priori* subgroup analyses, the risk of hospitalization in patients treated with a high dose PQ was also lower than that in patients not treated with PQ (Table 3).

### Risk of death

**Fig 4:**
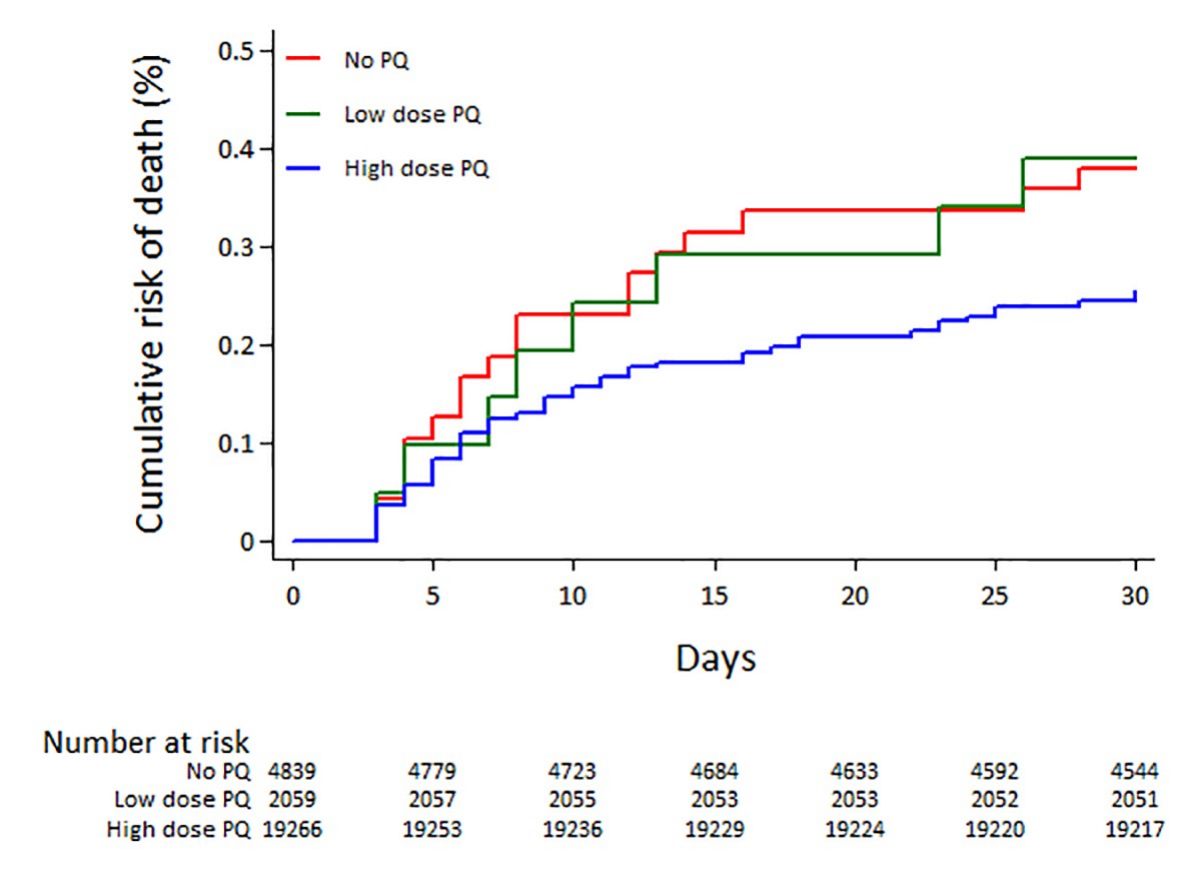
Risk of all cause death by primaquine (PQ) dose.

A total of 75 (0.28%) patients died between day 3 and 30 days, of whom 18 (24.00%) were not treated with PQ, 8 (10.67%) were treated with low PQ and 49 (65.33%) were treated with high dose PQ. The median number of days before death was 8 (IQR: 5–16; range 3–30) and did not vary with PQ treatment (p = 0.887). The cumulative risk of death at 30 days was 0.27% (95%CI: 0.21–0.35) in those treated with any dose of PQ and 0.38% (95%CI: 0.24–0.60) in patients not treated with PQ; AHR = 0.79 (95%CI: 0.43–1.45); p = 0.448. The rate of death was not significantly lower in patients treated with high dose PQ; AHR = 0.77 (95%CI: 0.41–1.43); p = 0.403 (Fig 4, Table 2). Baseline risk factors for death are presented in S4 Table. In the *a priori* subgroup analyses, the rate of death in non-Papuans after high dose PQ was significantly lower than that in patients not treated with PQ; AHR = 0.05 (95%CI: 0.006–0.43); p = 0.006 (Table 3).

At time of death 50 (66.67%) of 75 patients had represented with malaria: 5 (10.00%) with *P*. *falciparum*, 26 (52.00%) with *P*. *vivax* infection and 19 (38.00%) with had a mixed species infection. Of those who died a total of 4 (5.33%) were diagnosed with acute renal failure, three of whom had received high dose PQ and one didn’t receive any PQ. The clinical details of the 75 patients who died are summarized in S5 Table.

### Haematological outcomes

Data on full blood count were available in 8,964 (34.19%) patients at their initial presentation, of whom 20.25% (1,815) had a subsequent Hb measure between day 3 and 30. Among those a total of 253 (13.94%) patients presented with an Hb below 7g/dl at first visit. The risk of the Hb concentration falling below 7g/dl was 14.15% (95%CI: 12.45–16.05) in patients treated with any dose PQ compared to 20.43% (95%CI: 16.67–24.89) in those not treated with PQ; AHR = 0.66 (95%CI: 0.45–0.97); p = 0.033. Overall the NNT with PQ to prevent one patient dropping their Hb below 7g/dl within 30 days was 16 (95%CI: 9–183). The comparative AHR for PQ treatment versus those not treated with PQ for the Hb falling below 7g/dl before day 14 were 0.61 (95%CI: 0.39–0.97; p = 0.036), and between day 15 and 30 it was 0.75 (95%CI: 0.38–1.45; p = 0.385) (Table 4). The trends for the outcome of Hb falling below 5 g/dl were similar (Table 4).

**Table 4:** Risk of anaemia within 30 days after any PQ dose and no PQ.

			N	Number of events	Cumulative risk in % (95%CI)	Adjusted Hazard ratio (95%CI)^1^	p
**Hb falling below 7g/dl**	Day 3–30	No PQ	373	76	20.43 (16.67–24.89)	Reference	
		Low dose PQ	143	29	20.28 (14.56–27.85)	0.93 (0.54–1.58)	0.776
		High dose PQ	1,299	175	13.47 (11.73–15.45)	0.62 (0.42–0.91)	0.015
		Any PQ	1,442	204	14.15 (12.45–16.05)	0.66 (0.45–0.97)	0.033
	Day 3–14	No PQ	373	42	11.26 (8.45–14.93)	Reference	
		Low dose PQ	143	14	9.79 (5.92–15.97)	0.81 (0.40–1.61)	0.541
		High dose PQ	1,299	144	8.78 (7.36–10.45)	0.58 (0.36–0.93)	0.024
		Any PQ	1,442	128	8.88 (7.52–10.47)	0.61 (0.39–0.97)	0.036
	Day 15–30	No PQ	373	34	10.33 (7.49–14.15)	Reference	
		Low dose PQ	143	15	11.63 (7.18–18.54)	1.12 (0.48–2.63)	0.796
		High dose PQ	1,299	61	5.15 (4.03–6.57)	0.66 (0.33–1.31)	0.232
		Any PQ	1,442	76	5.78 (4.65–7.19)	0.75 (0.38–1.45)	0.385
**Hb falling below 5g/dl**	Day 3–30	No PQ	373	20	5.37 (3.50–8.20)	Reference	
		Low dose PQ	143	8	5.59 (2.84–10.87)	1.21 (0.44–3.32)	0.716
		High dose PQ	1,299	44	3.39 (2.53–4.52)	0.91 (0.43–1.96)	0.818
		Any PQ	1,442	52	3.61 (2.76–4.71)	0.97 (0.46–2.03)	0.933
	Day 3–14	No PQ	373	13	3.49 (2.04–5.93)	Reference	
		Low dose PQ	143	4	2.80 (1.06–7.28)	1.05 (0.29–3.76)	0.938
		High dose PQ	1,299	28	2.16 (1.49–3.11)	0.77 (0.31–1.89)	0.567
		Any PQ	1,442	32	2.22 (1.57–3.12)	0.81 (0.33–1.95)	0.641
	Day 15–30	No PQ	373	7	1.95 (0.94–4.06)	Reference	
		Low dose PQ	143	4	2.88 (1.09–7.49)	1.53 (0.28–8.23)	0.620
		High dose PQ	1,299	16	1.26 (0.77–2.05)	1.27 (0.30–5.44)	0.744
		Any PQ	1,442	20	1.42 (0.92–2.19)	1.35 (0.34–5.38)	0.672
**Hb falling more than 25% to below 7g/dl**	Day 3–30	No PQ	188	7	3.76 (1.81–7.72)	Reference	
		Low dose PQ	79	5	6.33 (2.68–14.54)	1.70 (0.48–6.06)	0.415
		High dose PQ	637	24	3.77 (2.55–5.57)	1.05 (0.39–2.87)	0.919
		Any PQ	716	29	4.05 (2.83–5.78)	1.16 (0.43–3.10)	0.769
	Day 3–14	No PQ	188	3	1.60 (0.52–4.87)	Reference	
		Low dose PQ	79	1	1.27 (0.18–8.65)	1.16 (0.94–14.18)	0.910
		High dose PQ	637	6	0.94 (0.42–2.08)	1.02 (0.16–2.6)	0.986
		Any PQ	716	7	0.98 (0.47–2.04)	1.04 (0.19–5.80)	0.962
	Day 15–30	No PQ	188	4	2.20 (0.83–5.75)	Reference	
		Low dose PQ	79	4	5.13 (1.96–13.09)	2.06 (0.46–9.29)	0.349
		High dose PQ	637	18	2.85 (1.81–4.49)	1.11 (0.32–3.84)	0.870
		Any PQ	716	22	3.10 (2.05–4.67)	1.27 (0.37–4.34)	0.706

Abbreviations: PQ = primaquine

^1^ Cox model stratified by year, and adjusted for sex, ethnicity, admission status and age, including an interaction term for age and baseline haemoglobin.

A total 36 patients (3.98%; 36/904) had a drop in Hb greater than 25% to an absolute Hb <7g/dL within the first 30 days. These included 4.05% (29/716) of patients treated with PQ, and 3.72% (7/188) of patients not receiving PQ. In the multivariable analysis there was no difference in the rate of a clinically significant fall in Hb between those treated with PQ and those who were not administered PQ; AHR = 1.16 (95%CI: 0.43–3.10); p = 0.769 (Table 4). The clinical details of those 36 patients are summarized in S6 Table.

## Discussion

Our study quantified the morbidity and mortality in more than 26,000 patients presenting with vivax malaria treated with or without PQ in a routine healthcare setting. Although patients were prescribed PQ according to local guidelines, without prior testing for G6PD deficiency, patients treated with PQ had a significantly lower risk of re-presenting to hospital, admission to hospital and developing severe anaemia compared to those patients not treated with PQ within 30 days. The overall risk of dying was lower after high dose PQ, although confidence intervals were wide.

Current WHO treatment guidelines recommend a 14 day course of PQ at 0.25mg/kg/day, but also state that a higher dose (0.5mg/kg/day) may be required in areas with high *P*. *vivax* relapse periodicity such as East Asia and Oceania [7]. In Papua the treatment guidelines changed in March 2006 from the lower dose to the higher dose regimen, but since G6PD testing was not available, patients were treated without prior exclusion of G6PD deficiency.

Providing safe and effective radical cure is important at both an individual and community level. Recurrent episodes of vivax malaria result in a cumulative risk of severe anaemia and associated morbidity and mortality [1,19]. They also contribute to onwards transmission, undermining efforts to eliminate malaria [20,21]. This has to be balanced against the risk of PQ-induced haemolysis and other severe adverse events [22,23]. PQ radical cure is recommended in the antimalarial treatment guidelines of most malaria endemic countries, but in practice its prescription is often low, since policy makers and healthcare providers are reluctant to use it, primarily due to fears of toxicity in G6PD deficient patients [5,24].

Our analysis shows that in Papua, Indonesia, providing PQ radical cure without prior testing for G6PD reduced the risks of re-presentation within 30 days. A previous analysis from the same location also showed a modest, but statistically significant, reduction in *P*. *vivax* re-presentations in the year following initial presentation [14]. In the current study high dose PQ was also associated with a 33% reduction in the risk of anaemia after treatment and approximately half the risk of all cause hospitalization within 30 days. The reduction in anaemia likely reflects prevention of additive haematological insult of recurrent vivax malaria. The reduction of all-cause hospitalization if confirmed, argues that reduction in *P*. *vivax* relapses by the provision of effective radical cure would also reduce vulnerability to other non-malaria illnesses [12,19,25]. Reassuringly, we observed no overall increased risk of mortality or massive Hb reductions in patients treated with PQ.

Our study has a number of strengths. The very large sample size reduces the possibility of chance findings. Furthermore, the patients included in this analysis represent a sample of the population seeking treatment for vivax malaria in a real world setting as compared to carefully selected patient cohorts enrolled into comparative clinical trials. This increases the applicability of the results to the patient population of interest.

Our study also has a number of important limitations. The decision regarding whether or not to prescribe PQ was made by the attending clinician and patients were not allocated randomly to a treatment regimen. Although clinicians may have been hesitant to prescribe PQ to patients who were very unwell or had significant anaemia, discussions with senior clinicians at the hospital suggest that omission of PQ was more often related to junior doctors being less aware of the need to offer PQ radical cure. The baseline characteristics between the treatment groups were generally comparable, however inpatients were less likely to receive PQ than outpatients, but are known to have a higher risk of re-presentation, hospitalization and death. In the *a priori* subgroup analysis in which only outpatients were included, the risk of re-presentation was also lower in the PQ group. The remaining *a priori* subgroup analyses confirmed similar trends in reduction of the primary and secondary outcomes. The subgroup of patients presenting in 2007, was to include a pharmacy stock-out of PQ which lasted for 7 months, with patients also included from 6 months intervals before and after this period. The objective was to focus on patients who didn’t receive PQ due to logistical rather than clinical reasons. Whilst the trend for reduction in re-presentation and hospital admission remained, the confidence intervals were wide due to reduced sample size.

In our analysis, patients with an event on day 1 or 2 were censored, assuming that these are related to deterioration from clinical malaria rather than its treatment. In a recent meta-analysis the nadir Hb in patients with uncomplicated *P*. *vivax* malaria occurred on day 2, even in patients not treated with PQ and thus is likely to be predominantly related to malaria [26]. Reports of severe haemolytic adverse events attributable to PQ suggest that these present between day 3 and day 14 [23,27]. However, it is possible that our approach may have missed some early serious adverse events due to PQ, particularly those related to gastrointestinal upset.

A further limitation of the study was that it was not possible to confirm from the hospital records whether patients took the full course of PQ treatment or not. Previously we have postulated that poor adherence to PQ treatment at the same location may limit effectiveness of radical cure [14]. The modest number of serious adverse outcomes might therefore be attributable to patients not adhering to a full course of treatment. A similar safety analysis of infants in the same setting highlighted that almost 19% of patients treated with high dose PQ had severe anaemia during follow up compared to less than 5% in infants not treated with PQ [28]. The greater risk of anaemia in these infants could be due to higher adherence rates encouraged by parents and caregivers. Our study relied on passive follow up, assuming that most patients would re-present to the same hospital if they became unwell. Until early 2010, the RSMM was the only tertiary health facility and healthcare for most patients is free, thus most patients return to the hospital for ongoing care [29]. Although we cannot rule out attrition bias, we assumed the degree of bias to be similar in patients who did and did not receive PQ.

Our analysis included all severe adverse events within 3 to 30 days of patients’ initial presentation with vivax malaria. Hb concentration was not measured routinely, although it is hospital practice to check the Hb of inpatients and those with clinical anaemia. Our primary analysis therefore focused on all cause re-presentation and hospital admission as indirect measures of adverse outcomes rather than direct measures of haemolysis. The latter was addressed in a subgroup of patients in whom Hb was measured at presentation, and reassuringly showed similar trends to a lower risk of anaemia in patients receiving PQ compared to those who did not.

We were also unable to discriminate whether the reported clinical endpoints were attributable to malaria, non-malaria or drug-induced aetiologies. It is possible that some of the severe adverse events could have been due to PQ-induced toxicity in G6PD deficient individuals, and that these could have been prevented if prior G6PD testing had been offered. Whilst all efforts should be made to diagnose patients with G6PD deficiency prior to PQ administration, this is often unavailable or unreliable due to logistical, financial and infrastructural limitations in remote and inaccessible locations where the main burden of malaria resides [2,5]. The options in these circumstances include prohibiting the use of PQ completely or administering the drug with additional measures to mitigate severe adverse reactions by detecting early signs of haemolysis and ceasing further drug intake. The consequences of not prescribing PQ must be weighed carefully against the considerable, and often neglected, benefits of preventing recurrent *P*. *vivax* infection and its associated morbidity and mortality. Our analysis in Papua, suggests that irrespective of the aetiology, one re-presentation to hospital can be prevented by treating 24 patients with PQ even without G6PD testing. The corresponding NNT with PQ was 16 to prevent one patient dropping their Hb below 7g/dl and 81 to prevent one patient requiring admission to hospital.

The relevance of our findings for patients with vivax malaria in other endemic regions needs to be considered with caution. In southern Papua less than 3% of the population are G6PD deficient [8], a likely reflection of the high proportion of the population who are of Highland ethnicity and therefore whose ancestors will have lived in non-malaria areas. The observed low risks associated with PQ, cannot be extrapolated to areas with a higher prevalence of G6PD deficiency or other haemoglobinopathies or red cell variants. In conclusion our analysis highlights that whilst it is important to ensure that PQ be administered safely, clinicians and policy makers must take into consideration both the risk of severe adverse events as well as the potential substantial and often neglected benefits of reducing the early recurrence of malaria and its associated direct and indirect morbidity and mortality [12]. In Papua our analysis provides reassuring evidence from a routine care setting, that the beneficial effects of PQ, even without prior G6PD testing, outweigh its risks.

## Supporting information

S1 Text Statistical Analysis Plan. (PDF)

S1 Data Data file. (CSV)

S1 Table RECORD checklist. (PDF)

S2 Table Baseline risk factors for any re-presentation to hospital within 30 days after treatment with different doses of primaquine. (PDF)

S3 Table Baseline risk factors for hospitalization to hospital within 30 days after treatment with different doses of primaquine. (PDF)

S4 Table Baseline risk factors for dying within 30 days after treatment with different doses of primaquine. (PDF)

S5 Table Haematological and clinical details of the 75 patients who died. (PDF)

S6 Table Haematological and clinical details of the 36 patients whose Hb fell by >25% to below 7g/dl. (PDF)

## References

[1] Douglas NM, Anstey NM, Buffet PA, Poespoprodjo JR, Yeo TW, White NJ, et al The anaemia of Plasmodium vivax malaria. Malaria journal. 2012;11:135 10.1186/1475-2875-11-135 22540175 PMC3438072

[2] Price RN, Commons RJ, Battle KE, Thriemer K, Mendis K. Plasmodium vivax in the Era of the Shrinking P. falciparum Map. Trends in parasitology. 2020;36(6):560–70. 10.1016/j.pt.2020.03.009 . 32407682 PMC7297627

[3] Anstey NM, Russell B, Yeo TW, Price RN. The pathophysiology of vivax malaria. Trends in parasitology. 2009;25(5):220–7. 10.1016/j.pt.2009.02.003 . 19349210

[4] White MT, Shirreff G, Karl S, Ghani AC, Mueller I. Variation in relapse frequency and the transmission potential of Plasmodium vivax malaria. Proc Biol Sci. 2016;283(1827):20160048 10.1098/rspb.2016.0048 27030414 PMC4822465

[5] Thriemer K, Ley B, Bobogare A, Dysoley L, Alam MS, Pasaribu AP, et al Challenges for achieving safe and effective radical cure of Plasmodium vivax: a round table discussion of the APMEN Vivax Working Group. Malaria journal. 2017;16(1):141 10.1186/s12936-017-1784-1 28381261 PMC5382417

[6] von Seidlein L, Auburn S, Espino F, Shanks D, Cheng Q, McCarthy J, et al Review of key knowledge gaps in glucose-6-phosphate dehydrogenase deficiency detection with regard to the safe clinical deployment of 8-aminoquinoline treatment regimens: a workshop report. Malaria journal. 2013;12:112 10.1186/1475-2875-12-112 23537118 PMC3616837

[7] WHO. Guidelines for the treatment of malaria 2015 Available from: http://apps.who.int/iris/bitstream/10665/162441/1/9789241549127_eng.pdf?ua=1&ua=1.

[8] Pava Z, Burdam FH, Handayuni I, Trianty L, Utami RA, Tirta YK, et al Submicroscopic and Asymptomatic Plasmodium Parasitaemia Associated with Significant Risk of Anaemia in Papua, Indonesia. PloS one. 2016;11(10):e0165340 10.1371/journal.pone.0165340 27788243 PMC5082812

[9] Kenangalem E, Poespoprodjo JR, Douglas NM, Burdam FH, Gdeumana K, Chalfein F, et al Malaria morbidity and mortality following introduction of a universal policy of artemisinin-based treatment for malaria in Papua, Indonesia: A longitudinal surveillance study. PLoS medicine. 2019;16(5):e1002815 10.1371/journal.pmed.1002815 31167228 PMC6541239

[10] Karyana M, Devine A, Kenangalem E, Burdarm L, Poespoprodjo JR, Vemuri R, et al Treatment-seeking behaviour and associated costs for malaria in Papua, Indonesia. Malaria journal. 2016;15(1):536 10.1186/s12936-016-1588-8 27821127 PMC5100266

[11] Douglas NM, Lampah DA, Kenangalem E, Simpson JA, Poespoprodjo JR, Sugiarto P, et al Major burden of severe anemia from non-falciparum malaria species in Southern Papua: a hospital-based surveillance study. PLoS medicine. 2013;10(12):e1001575; discussion e. 10.1371/journal.pmed.1001575 24358031 PMC3866090

[12] Dini S, Douglas NM, Poespoprodjo JR, Kenangalem E, Sugiarto P, Plumb ID, et al The risk of morbidity and mortality following recurrent malaria in Papua, Indonesia: a retrospective cohort study. BMC medicine. 2020;18(1):28 10.1186/s12916-020-1497-0 32075649 PMC7031957

[13] Karyana M, Burdarm L, Yeung S, Kenangalem E, Wariker N, Maristela R, et al Malaria morbidity in Papua Indonesia, an area with multidrug resistant Plasmodium vivax and Plasmodium falciparum. Malar J. 2008;7:148 10.1186/1475-2875-7-148 . 18673572 PMC2518158

[14] Douglas NM, Poespoprodjo JR, Patriani D, Malloy MJ, Kenangalem E, Sugiarto P, et al Unsupervised primaquine for the treatment of Plasmodium vivax malaria relapses in southern Papua: A hospital-based cohort study. PLoS medicine. 2017;14(8):e1002379 10.1371/journal.pmed.1002379 28850568 PMC5574534

[15] Ratcliff A, Siswantoro H, Kenangalem E, Wuwung M, Brockman A, Edstein MD, et al Therapeutic response of multidrug-resistant Plasmodium falciparum and P. vivax to chloroquine and sulfadoxine-pyrimethamine in southern Papua, Indonesia. Transactions of the Royal Society of Tropical Medicine and Hygiene. 2007;101(4):351–9. 10.1016/j.trstmh.2006.06.008 17028048 PMC2080856

[16] Ratcliff A, Siswantoro H, Kenangalem E, Maristela R, Wuwung RM, Laihad F, et al Two fixed-dose artemisinin combinations for drug-resistant falciparum and vivax malaria in Papua, Indonesia: an open-label randomised comparison. Lancet. 2007;369(9563):757–65. 10.1016/S0140-6736(07)60160-3 17336652 PMC2532500

[17] Hasugian AR, Purba HL, Kenangalem E, Wuwung RM, Ebsworth EP, Maristela R, et al Dihydroartemisinin-piperaquine versus artesunate-amodiaquine: superior efficacy and posttreatment prophylaxis against multidrug-resistant Plasmodium falciparum and Plasmodium vivax malaria. Clinical infectious diseases: an official publication of the Infectious Diseases Society of America. 2007;44(8):1067–74. 10.1086/512677 17366451 PMC2532501

[18] Altman DG, Andersen PK. Calculating the number needed to treat for trials where the outcome is time to an event. Bmj. 1999;319(7223):1492–5. 10.1136/bmj.319.7223.1492 10582940 PMC1117211

[19] Douglas NM, Pontororing GJ, Lampah DA, Yeo TW, Kenangalem E, Poespoprodjo JR, et al Mortality attributable to Plasmodium vivax malaria: a clinical audit from Papua, Indonesia. BMC medicine. 2014;12:217 10.1186/s12916-014-0217-z 25406857 PMC4264333

[20] Robinson LJ, Wampfler R, Betuela I, Karl S, White MT, Li Wai Suen CS, et al Strategies for Understanding and Reducing the Plasmodium vivax and Plasmodium ovale Hypnozoite Reservoir in Papua New Guinean Children: A Randomised Placebo-Controlled Trial and Mathematical Model. PLoS Med. 2015;12(10):e1001891 10.1371/journal.pmed.1001891 eCollection 2015 Oct. . 26505753 PMC4624431

[21] Douglas NM, Simpson JA, Phyo AP, Siswantoro H, Hasugian AR, Kenangalem E, et al Gametocyte dynamics and the role of drugs in reducing the transmission potential of Plasmodium vivax. The Journal of infectious diseases. 2013;208(5):801–12. 10.1093/infdis/jit261 23766527 PMC3733516

[22] Ashley EA, Recht J, White NJ. Primaquine: the risks and the benefits. Malaria journal. 2014;13:418 10.1186/1475-2875-13-418 25363455 PMC4230503

[23] Chu CS, Bancone G, Moore KA, Win HH, Thitipanawan N, Po C, et al Haemolysis in G6PD Heterozygous Females Treated with Primaquine for Plasmodium vivax Malaria: A Nested Cohort in a Trial of Radical Curative Regimens. PLoS medicine. 2017;14(2):e1002224 10.1371/journal.pmed.1002224 28170391 PMC5295665

[24] Ley B, Thriemer K, Jaswal J, Poirot E, Alam MS, Phru CS, et al Barriers to routine G6PD testing prior to treatment with primaquine. Malaria journal. 2017;16(1):329 10.1186/s12936-017-1981-y 28797255 PMC5553859

[25] Maitland K, Berkley JA, Shebbe M, Peshu N, English M, Newton CR. Children with severe malnutrition: can those at highest risk of death be identified with the WHO protocol? PLoS medicine. 2006;3(12):e500 10.1371/journal.pmed.0030500 17194194 PMC1716191

[26] Commons RJ, Simpson JA, Thriemer K, Chu CS, Douglas NM, Abreha T, et al The haematological consequences of Plasmodium vivax malaria after chloroquine treatment with and without primaquine: a WorldWide Antimalarial Resistance Network systematic review and individual patient data meta-analysis. BMC medicine. 2019;17(1):151 10.1186/s12916-019-1386-6 31366382 PMC6670141

[27] Taylor WRJ, Thriemer K, von Seidlein L, Yuentrakul P, Assawariyathipat T, Assefa A, et al Short-course primaquine for the radical cure of Plasmodium vivax malaria: a multicentre, randomised, placebo-controlled non-inferiority trial. Lancet. 2019 10.1016/S0140-6736(19)31285-1 31327563 PMC6753019

[28] Setyadi A, Arguni E, Kenangalem E, Hasanuddin A, Lampah DA, Thriemer K, et al Safety of primaquine in infants with Plasmodium vivax malaria in Papua, Indonesia. Malaria journal. 2019;18(1):111 10.1186/s12936-019-2745-7 30940140 PMC6444676

[29] Devine A, Kenangalem E, Burdam FH, Anstey NM, Poespoprodjo JR, Price RN, et al Treatment-Seeking Behavior after the Implementation of a Unified Policy of Dihydroartemisinin-Piperaquine for the Treatment of Uncomplicated Malaria in Papua, Indonesia. The American journal of tropical medicine and hygiene. 2018;98(2):543–50. 10.4269/ajtmh.17-0680 29280424 PMC5810904

