# Supplementary material for: The risk of adverse clinical outcomes following treatment of Plasmodium vivax malaria with and without primaquine in Papua, Indonesia
Source: PLoS Negl Trop Dis. 2020 Nov 11;14(11):e0008838. doi: 10.1371/journal.pntd.0008838 (PMC7657498; doi:10.1371/journal.pntd.0008838)
Supplement: S1 Text — (PDF) [file pntd.0008838.s001.pdf]

S1 Text: Statistical Analysis Plan

1. Objectives

To define the risk of serious adverse events following administration of unsupervised primaquine without prior G6PD testing

Research Question: Is the risk of serious adverse events after 14 days of unsupervised primaquine worse than that observed in those treated for *P. vivax* malaria without primaquine?

Outcomes of Interest: Risk of serious adverse events (admission to hospital, representation, death and severe anaemia) within 30 days of treatment of *P. vivax* infection.

2. Study population

Patients presenting to any department at Rumah Sakit Mitra Masyarakat with microscopically confirmed *P. vivax* parasitaemia (either as a mono-infection or part of a mixed infection) regardless of presence or absence of symptoms.

Exclusions:

- Clinical episodes not associated with parasitaemia or associated with infection by *Plasmodium* species other than *P. vivax*
- Clinical episodes not treated with an artemisinin combination therapy (ACT) as schizontocidal treatment or where schizontocidal treatment is unknown
- Clinical episodes that cannot be matched with antimalarial prescription records
- Clinical episodes in infants under the age of one year
- Clinical episodes in patients without age recorded
- Clinical episodes in pregnant women in any trimester

3. Endpoints:

- The cumulative risk of hospital admission within 30 days after initiation of treatment of *P. vivax* comparing the different primaquine (PQ) dosing arms (described below).
- The cumulative risk of death within 30 days after presentation with *P. vivax* comparing the different PQ dosing arms.
- The cumulative risk of representation within 30 days of treatment of *P. vivax* comparing the different PQ dosing arms.
- Subgroup analysis of individuals readmitted to hospital: risk of Hb falling below 7g/dl, risk of Hb falling below 5g/dl and risk of having a fractional Hb fall >25% to below 7g/dl
- Description of events at the time of death and identification of potential relationship to PQ dosing arms.

4. Definitions

4.1 PQ dosing categories:

Pharmacy records reported the number of tablets prescribed and the actual mg per kg dose of PQ prescribed was derived for each individual patient by applying estimated the mean body weights within each age, sex, and ethnicity strata derived from a cross sectional survey. Patients in whom pharmacy records could be matched with hospital records are then categorised into 5 groups:

a) No primaquine (matching antimalarial prescription data but no primaquine prescribed)
b) Single dose primaquine (>0mg/kg and <1.5mg/kg total dose)
c) Low dose primaquine (≥1.5mg/kg and <5mg/kg total dose)
d) High dose primaquine (≥5mg/kg total dose)
e) Unknown dose primaquine (matched primaquine prescription record but unable to determine dose in mg per kg)

4.2 Follow up:

All presentations to hospital within 14 days are assumed to be due to the same episode of malaria. Hence multiple presentations with *Plasmodium* parasitaemia within 14 days are concatenated into a single clinical episode.

4.3 Clinical Diagnoses:

Clinical diagnoses are defined by the attending clinician and recorded using the ICD classification. The hospital has 101 beds, a 24-hour emergency department, an active outpatients "polyclinic", and a high care unit with facilities for intravenous infusions but not mechanical ventilation. Radiological services include X-ray and ultrasonography. Laboratory facilities are also available for hematologic and biochemical analysis and basic microscopy, but not for microbiological culture.

4.4 Clinical Events Potentially Attributable to Primaquine toxicity:

Clinical events potentially attributable to primaquine toxicity will include: Severe anaemia, acute renal failure, haemoglobinuria, gastrointestinal symptoms.

5. Statistical methods

5.1. Baseline and demographic

A flow diagram will be provided of participants excluded and included in the analysis. Baseline data on all patients included in the analysis will be presented. Baseline data will be presented by PQ dosing group. Baseline data will be presented as N (%) and median (inter-quartile range (IQR)) as appropriate. Baseline variables included are sex, age, ethnic group, year of presentation, nutritional status, anaemia on admission and admission status.

5.2 The cumulative risk of representation within 30 days after treatment for *P. vivax*

The cumulative risk of representation will be assessed using survival analyses (Kaplan Meier) for all PQ groups comparing patients receiving any dose of PQ, low dose PQ, high dose PQ and those not prescribed PQ.

In the survival analyses, events will include representation between day 3 and day 30. Patients representing on day 1-3 will be censored on that day under the assumption that this hospital visit was related to the initial vivax episode rather than a new representation. Patients representing after day 30 will be censored and their last day of follow up be set as day 30.

Hazard Ratios will be calculated using a Cox regression model. A univariable analysis will be done for PQ dosing groups, sex, ethnic group, admission status, age group, baseline haemoglobin, and year of enrolment. Only the first episode per patient will be used for the univariable analysis. Multivariable analyses will be done including all variables from the univariable analyses and all episodes.

Pre-specified subgroup comparisons will be done using the final model for each analysis stratified by year (limited to high dose PQ and no PQ):

- Each age category (1 to <5 years, 5 to <15 years, ≥15 years)
- 12 months pre and post the largest primaquine stock outage in 2007
- Patients initially treated as outpatients (not applicable for analyses of risk of admission since this analysis by definition is restricted to outpatients)
- Patients of Lowland and non-Papuan ethnicity

5.3 The cumulative risk of admission within 30 days after treatment of *P. vivax*

The cumulative risk of admission will be assessed using survival analyses (Kaplan Meier) for all PQ groups comparing patients receiving any dose of PQ, low dose PQ, high dose PQ and those no prescribed PQ.

In the survival analyses, events will be defined in patients who were initially treated as an outpatient, but who required admission for inpatient care between 3 and 30 days of presentation. Patients admitted on day 1-3 will be censored on that day under the assumption that this admission was related to the initial vivax episode rather than a new admission. Patients admitted after day 30 will be censored and their last day of follow up be set as day 30.

Hazard Ratios will be calculated using a Cox regression model. Univariable and multivariable analyses as well as subgroup analyses will be the same as described in Sections 5.2.

5.4 The cumulative risk of mortality within 30 days after treatment for *P. vivax*

The cumulative risk of death will be assessed using survival analyses (Kaplan Meier) for all PQ groups comparing patients receiving any dose of PQ, low dose PQ, high dose PQ and those no prescribed PQ.

In the survival analyses, events will be defined in patients who died between 3 and 30 days of presentation. Patients dying on day 1-3 will be censored on that day under the assumption that this

admission was related to the initial vivax episode rather than a new admission. Patients admitted after day 30 will be censored and their last day of follow up be set as day 30. Hazard Ratios will be calculated using a Cox regression model. Univariable and multivariable analyses as well as subgroup analyses will be the same as described in Sections 5.2.

5.5 Subgroup analyses of individuals readmitted to hospital risk of Hb falling below 7g/dl, risk of Hb falling below 5g/dl and risk of having a fractional Hb fall >25% to below 7g/dl

Absolute change of Hb and fractional change (%) will be calculated for each patient where both measurements are available on presentation and again on readmission to hospital.

The incidence risk of falling below 7g/dl and to 5g/dl between initial enrolment and readmission will be assessed using survival analyses (Kaplan Meier) comparing patients receiving any dose primaquine and those not prescribed PQ.

In the survival analyses, events will be defined in patients who had a Hb measurement below 7g/dl or 5 g/dl and between initial enrolment and readmission before day 30. Hazard Ratios will be calculated using a Cox regression model.

The incidence risk of having a fractional fall >25% to below 7g/dl in Hb between initial enrolment and readmission will be assessed using survival analyses (Kaplan Meier) comparing patients receiving any dose primaquine and those not prescribed PQ.

In the survival analyses, events will be defined in patients who had a fractional fall >25% in Hb to below 7g/dl measurement at enrolment and readmission. Hazard Ratios will be calculated using a Cox regression model.

5.5 Description of events between initial malaria and death

For patients who died within 30 days of initial treatment, all of the clinical diagnoses documented between the initial presentation and time of death will be reviewed. Individual diagnoses will be collated into those potentially attributable to primaquine induced toxicity. The frequency of potentially attributable and contributory factors will be presented according to age, ethnicity and the prior administered primaquine regimen.
