# Supplementary material for: The risk of adverse clinical outcomes following treatment of Plasmodium vivax malaria with and without primaquine in Papua, Indonesia
Source: PLoS Negl Trop Dis. 2020 Nov 11;14(11):e0008838. doi: 10.1371/journal.pntd.0008838 (PMC7657498; doi:10.1371/journal.pntd.0008838)
Supplement: S2 Table — (PDF) [file pntd.0008838.s004.pdf]

S2 Table. Baseline risk factors for any representation to hospital within 30 days after treatment with different doses of primaquine

	**Cumulative risk in % (95% CI)**	**Unadjusted Hazard Ratio (95% CI)**	**P**	**Adjusted Hazard Ratio[1] (95% CI)**	**P**
**Initial Species**					
Pure *P. vivax*	28.55 (27.90-29.21)	Reference		Reference	
Mixed *P. vivax/P. falciparum*	26.88 (25.91-27.88)	0.93 (0.89-0.98)	0.007	0.92 (1.01-1.17)	0.003
**Sex**					
Male	25.85 (25.13-26.60)	Reference		Reference	
Female	30.47 (29.67-31.29)	1.21 (1.16-1.27)	<0.0001	1.17 (1.11-1.22)	<0.0001
**Ethnicity**					
Non-Papuan	19.62 (18.43-20.87)	Reference		Reference	
Highland	30.47 (29.83-31.12)	1.63 (1.52-1.76)	<0.0001	1.64 (1.52-1.77)	<0.0001
Lowland	22.11 (20.42-23.91)	1.13 (1.01-1.27)	0.032	1.12 (1.00-1.26)	0.058
**Age**					
1 to <5 years	33.99 (32.71-35.30)	1.23 (1.17-1.30)	<0.0001	1.21 (1.14-1.28)	<0.0001
5 to <15 years	21.65 (20.49-22.87)	0.75 (0.70-0.80)	<0.0001	0.73 (0.68-0.78)	<0.0001
≥15 years	27.99 (27.31-28.69)	Reference		Reference	
**Admission Status**					
Outpatient	27.78 (27.21-28.36)	Reference		Reference	
Inpatient	30.45 (28.75-32.23)	1.09 (1.02-1.17)	0.017	1.09 (1.01-1.17)	0.023

[1] Cox model stratified by year and PQ treatment, and adjusted for species at enrolment, sex, ethnicity, admission status and age.
