# Supplementary material for: The risk of adverse clinical outcomes following treatment of Plasmodium vivax malaria with and without primaquine in Papua, Indonesia
Source: PLoS Negl Trop Dis. 2020 Nov 11;14(11):e0008838. doi: 10.1371/journal.pntd.0008838 (PMC7657498; doi:10.1371/journal.pntd.0008838)
Supplement: S3 Table — (PDF) [file pntd.0008838.s005.pdf]

S3 Table. Baseline risk factors for hospitalization within 30 days after treatment with different doses of primaquine in patients initially treated as outpatients

	**Cumulative risk in % (95% CI)**	**Unadjusted Hazard Ratio (95% CI)**	**P**	**Adjusted Hazard Ratio[1] (95% CI)**	**P**
**Initial Species**					
Pure *P. vivax*	1.81 (1.62-2.02)	Reference		Reference	
Mixed *P. vivax/P. falciparum*	1.98 (1.68-2.35)	1.10 (0.89-1.35)	0.364	1.13 (0.91-1.39)	0.275
**Sex**					
Male	1.66 (1.45-1.90)	Reference		Reference	
Female	2.08 (1.83-2.36)	1.25 (1.04-1.51)	0.019	1.16 (0.96-1.40)	0.128
**Ethnicity**					
Non-Papuan	0.86 (0.60-1.22)	Reference		Reference	
Highland	2.09 (1.89-2.31)	2.45 (1.69-3.56)	<0.005	2.22 (1.52-2.24)	<0.001
Lowland	1.55 (1.09-2.21)	1.82 (1.10-3.02)	0.020	1.50 (0.90-2.52)	0.122
**Age**					
1 to <5 years	3.25 (2.77-3.80)	1.95 (1.59-2.40)	<0.005	1.90 (1.53-2.35)	<0.001
5 to <15 years	1.01 (0.75-1.36)	0.60 (0.43-0.83)	0.002	0.59 (0.43-0.82)	0.001
≥15 years	1.67 (1.48-1.90)	Reference		Reference	

[1] Cox model stratified by year and PQ treatment and adjusted for species at enrolment, sex, ethnicity and age.
