# Supplementary material for: The risk of adverse clinical outcomes following treatment of Plasmodium vivax malaria with and without primaquine in Papua, Indonesia
Source: PLoS Negl Trop Dis. 2020 Nov 11;14(11):e0008838. doi: 10.1371/journal.pntd.0008838 (PMC7657498; doi:10.1371/journal.pntd.0008838)
Supplement: S4 Table — (PDF) [file pntd.0008838.s006.pdf]

S4 Table. Baseline risk factors for dying within 30 days after treatment with different doses of primaquine

	**Cumulative risk in % (95% CI)**	**Unadjusted Hazard Ratio (95% CI)**	**P**	**Adjusted Hazard Ratio[1] (95% CI)**	**P**
**Initial Species**					
Pure *P. vivax*	0.26 (0.19-0.34)	Reference		Reference	
Mixed *P. vivax/P. falciparum*	0.36 (0.25-0.52)	1.39 (0.87-2.23)	0.163	0.95 (0.58-1.55)	0.847
**Sex**					
Male	0.37 (0.28-0.48)	Reference		Reference	
Female	0.20 (0.14-0.30)	0.55 (0.34-0.89)	0.015	0.53 (0.33-0.87)	0.001
**Ethnicity**					
Non-Papuan	0.27 (0.15-0.49)	Reference		Reference	
Highland	0.26 (0.20-0.35)	0.98 (0.51-1.87)	0.944	1.53 (0.78-3.00)	0.212
Lowland	0.55 (0.31-0.96)	2.04 (0.90-4.62)	0.088	3.22 (1.38-7.56)	0.007
**Age**					
1 to <5 years	0.27 (0.16-0.46)	0.77 (0.43-1.39)	0.392	0.63 (0.34-1.16)	0.137
5 to <15 years	0.09 (0.03-0.23)	0.25 (0.09-0.68)	0.007	0.27 (0.10-0.75)	0.012
≥15 years	0.35 (0.27-0.45)	Reference		Reference	
**Admission Status**					
Outpatient	0.11 (0.07-0.16)	Reference		Reference	
Inpatient	1.83 (1.39-2.40)	17.22 (10.66-27.83)	<0.0001	16.62 (10.09-27.35)	<0.0001

[1] Cox model stratified by year and PQ treatment and adjusted for species at enrolment, sex, ethnicity, age, and admission status
