# Supplementary material for: The risk of adverse clinical outcomes following treatment of Plasmodium vivax malaria with and without primaquine in Papua, Indonesia
Source: PLoS Negl Trop Dis. 2020 Nov 11;14(11):e0008838. doi: 10.1371/journal.pntd.0008838 (PMC7657498; doi:10.1371/journal.pntd.0008838)
Supplement: S5 Table — (PDF) [file pntd.0008838.s007.pdf]

S5 Table: Haematological and clinical details of the 75 patients who died

Patient number	Age category	Sex	Ethnic group	Malaria species at presentation	Hb (g/dl) at initial presentation	Lowest Hb (g/dl) during follow up	Number of days between presentation and lowest Hb	Number of days between presentation and death	Schizontocidal drugs	PQ dose	Clinical diagnosis assigned between presentation and death
1	>=15yr	M	LP	*P. vivax*	10.5	.	.	3	IV artesunate	High dose	Septic shock
											Acute MI
											Pericardial disease
											Bronchopneumonia
											COPD
2	>=15yr	M	HP	Mixed species	4.1	5.1	24	30	IV artesunate / DHA+PIP	High dose	Hypertension
											Chronic renal failure
											Pancytopaenia
											Pulmonary oedema
3	>=15yr	F	HP	Mixed species	3	.	.	6	IV artesunate / DHA+PIP	High dose	-
4	>=15yr	M	HP	*P. vivax*	.	4.7	6	11	DHA+PIP	High dose	Chronic bronchitis
											Chronic renal failure
											Hepatitis C
											HIV
5	>=15yr	M	HP	Mixed species	8.9	.	.	3	IV artesunate / DHA+PIP	High dose	COPD
											Chronic renal failure
											Supraventricular tachycardia
6	>=15yr	M	HP	Mixed species	5.3	.	.	5	IV artesunate / DHA+PIP	High dose	Hypertension
											Congestive heart failure
											Unstable angina
7	>=15yr	M	HP	*P. vivax*	7.8	5.2	6	23	DHA+PIP	High dose	Pneumonia
8	>=15yr	M	HP	*P. vivax*	.	11.6	18	25	DHA+PIP	High dose	Hepatitis B
											Hepatic failure

9	>=15yr	M	LP	*P. vivax*	14.2	15.4	9	11	DHA+PIP	High dose	Bronchitis
											Somatoform disorder
											Asthma
											Pulmonary tuberculosis
											Nontraumatic intracranial haemorrhage
											Conjunctivitis
10	>=15yr	M	LP	*P. vivax*	4.5	3.2	4	5	DHA+PIP	High dose	Chronic renal failure
											Essential hypertension
											Pneumonia
											Secondary hypertension
											Chronic renal failure
											Calcification of lung
11	>=15yr	M	LP	Mixed species	.	.	.	3	DHA+PIP	High dose	No other diagnostic information available
12	>=15yr	M	NP	Mixed species	15.1	.	.	5	IV artesunate	Nil	Septic shock
											Toxoplasmosis
											Bacterial meningitis
13	5-15yr	M	LP	*P. vivax*	9.5	11.4	18	28	Artesunate +amodiaquine	Nil	Protein-energy malnutrition
											Suspected tuberculosis
											Gastroenteritis
											Bacterial meningitis
14	>=15yr	M	LP	*P. vivax*	9.5	.	.	10	DHA+PIP	High dose	Pulmonary tuberculosis
											Tuberculous meningitis
15	>=15yr	M	HP	*P. vivax*	11.2	8.5	19	24	DHA+PIP	High dose	Acute upper respiratory infection
											Lobar pneumonia

											Gastroenteritis and colitis
											Septic shock
											Dehydration
16	>=15yr	M	HP	Mixed species	10.8	10.4	8	12	DHA+PIP	High dose	COPD
											Septic shock
											Lobar pneumonia
17	>=15yr	M	NP	*P. vivax*	5.3	5.3	3	3	IV artesunate / DHA+PIP / clindamycin	Nil	Diabetes mellitus
											Gangrenous ulcer
18	>=15yr	F	HP	Mixed species	2.5	.	.	12	IV artesunate / DHA+PIP	Nil	Pneumonia
											Bacterial meningitis
19	>=15yr	M	LP	Mixed species	11.1	11.6	8	9	DHA+PIP	High dose	Methanol poisoning
20	>=15yr	M	HP	*P. vivax*	9.1	.	.	4	IV artesunate / DHA+PIP	High dose	Septic shock
											Miliary tuberculosis
21	>=15yr	M	HP	*P. vivax*	.	4.8	3	22	DHA+PIP	High dose	Liver cirrhosis
											Acute cholecystitis
22	>=15yr	F	HP	*P. vivax*	3.5	.	.	23	IV artesunate / DHA+PIP	High dose	Chronic renal failure
											Hypovolaemic shock
											Iron deficiency anaemia
23	1-5yr	M	HP	*P. vivax*	12.2	.	.	4	IV artesunate	Nil	Tuberculous meningitis
											Marasmus
24	1-5yr	M	HP	Mixed species	.	.	.	18	DHA+PIP	High dose	Acute upper respiratory infection
											Drowning & submersion following fall into natural water
											Unattended death

25	>=15yr	M	NP	Mixed species	8	.	.	3	IV artesunate / clindamycin	Nil	-
26	>=15yr	M	HP	*P. vivax*	9	.	.	3	IV artesunate	High dose	Intracranial space-occupying lesion
27	>=15yr	M	HP	Mixed species	3.5	.	.	3	IV artesunate	Low dose	Chronic renal failure
28	>=15yr	M	HP	*P. vivax*	11.6	.	.	9	IV artesunate / DHA+PIP	High dose	Tuberculous meningitis
29	>=15yr	F	NP	*P. vivax*	10.1	.	.	4	IV artesunate / DHA+PIP	Nil	Intracerebral haemorrhage
30	1-5yr	M	HP	*P. vivax*	9	.	.	5	IV artesunate / DHA+PIP	High dose	Miliary tuberculosis
											Kwashiorkor
											Marasmus
31	1-5yr	M	LP	*P. vivax*	5.4	.	.	10	IV artesunate / DHA+PIP	High dose	Septic shock
											Trichuriasis
											Protein-energy malnutrition
											Gastroenteritis
32	>=15yr	M	HP	Mixed species	9.9	.	.	3	IV artesunate	High dose	Pneumonia
											Meningitis
											HIV
											Unattended death
33	>=15yr	F	HP	*P. vivax*	12	.	.	4	IV artesunate	High dose	Tuberculous meningitis
34	1-5yr	F	HP	Mixed species	6.8	4.1	27	30	DHA+PIP	High dose	Gastroenteritis
											Severe protein-energy malnutrition
35	>=15yr	F	HP	*P. vivax*	8.5	8	4	26	DHA+PIP	Nil	Postpartum haemorrhage
36	>=15yr	M	NP	*P. vivax*	11.8	.	.	4	DHA+PIP	Low dose	Meningitis
											Cardiac arrest, unspecified
37	1-5yr	F	HP	Mixed species	9.9	7.4	9	12	Artesunate +amodiaquine	Nil	Gastroenteritis

											Kwashiorkor
											Marasmus
											Pulmonary tuberculosis
38	>=15yr	M	HP	Mixed species	8	.	.	7	IV artesunate / DHA+PIP	High dose	Pulmonary tuberculosis
											Acute renal failure
											Urinary tract infection
39	1-5yr	M	HP	Mixed species	2.4	.	.	3	IV artesunate / DHA+PIP	High dose	Hereditary haemolytic anaemia
40	>=15yr	F	HP	*P. vivax*	6.6	8.7	7	7	DHA+PIP	High dose	Septicaemia
41	>=15yr	M	NP	Mixed species	10.2	.	.	13	Artesunate +amodiaquine	Nil	Pulmonary tuberculosis
42	>=15yr	F	HP	*P. vivax*	4.1	.	.	6	IV artesunate / DHA+PIP	High dose	Trichomoniasis
											Ischaemic cardiomyopathy
											Urinary tract infection
											Bronchitis
43	>=15yr	F	NP	*P. vivax*	6.3	.	.	13	IV artesunate / DHA+PIP	Low dose	HIV
											Tuberculosis
											Urinary tract infection
44	>=15yr	M	HP	*P. vivax*	.	14.8	19	25	DHA+PIP	High dose	Chronic bronchitis
											COPD
											Pneumonia
45	>=15yr	M	HP	*P. vivax*	11.5	.	.	6	IV artesunate / DHA+PIP	High dose	Meningitis
											Urinary tract infection
46	>=15yr	M	HP	*P. vivax*	3.9	.	.	5	IV artesunate / DHA+PIP	High dose	Postprocedural disorder of the nervous system not further specified

47	>=15yr	M	HP	*P. vivax*	.	8.4	12	16	DHA+PIP	High dose	Tuberculous meningitis
48	1-5yr	M	LP	*P. vivax*	.	7.2	7	7	DHA+PIP	Low dose	Marasmus
											Suspected tuberculosis
											Septicaemia
49	>=15yr	M	HP	Mixed species	12	.	.	6	IV artesunate / DHA+PIP	Nil	Primary cardiomyopathy
											Acute renal failure
											Bronchitis
50	5-15yr	F	NP	*P. vivax*	2.1	.	.	8	IV artesunate / DHA+PIP	Nil	Unattended death
51	>=15yr	F	NP	Mixed species	13.7	.	.	8	IV artesunate / DHA+PIP	High dose	-
52	1-5yr	M	HP	*P. vivax*	.	10.9	7	16	IV artesunate / DHA+PIP	Nil	Pulmonary tuberculosis
											Emphysema
											Measles
53	>=15yr	F	HP	*P. vivax*	8.7	.	.	17	IV artesunate / DHA+PIP	High dose	Essential hypertension
											Tuberculous peritonitis
											Unattended death
54	>=15yr	M	HP	*P. vivax*	8.9	.	.	10	DHA+PIP	Low dose	Stroke
											Bronchopneumonia
55	>=15yr	F	HP	*P. vivax*	2.6	.	.	4	IV artesunate	High dose	Septic shock
											Acute renal failure with tubular necrosis
											Chronic renal failure
56	1-5yr	F	HP	*P. vivax*	9.6	.	.	8	IV artesunate / DHA+PIP	Nil	Tuberculous meningitis
57	1-5yr	M	HP	*P. vivax*	.	9.4	10	14	DHA+PIP	Nil	Gastroenteritis
											Dehydration
											Pneumonitis due to food and vomit

58	>=15yr	F	HP	*P. vivax*	10.8	9.5	22	28	DHA+PIP	High dose	Intracranial space-occupying lesion
											Meningitis
59	>=15yr	F	HP	Mixed species	9.8	12.5	13	16	IV artesunate / DHA+PIP	High dose	Tuberculous meningitis
60	>=15yr	F	HP	*P. vivax*	11.1	.	.	8	IV artesunate	Low dose	Septic shock
											Meningitis
61	>=15yr	F	HP	*P. vivax*	10.6	.	.	5	IV artesunate	High dose	Meningitis
62	>=15yr	M	HP	Mixed species	13.1	.	.	6	IV artesunate	Nil	Miliary tuberculosis
											Tuberculous meningitis
63	>=15yr	F	LP	Mixed species	12	11.6	3	4	DHA+PIP	High dose	-
64	>=15yr	M	HP	*P. vivax*	6.5	.	.	6	IV artesunate / DHA+PIP / clindamycin	High dose	HIV
											Tuberculosis
											Acute renal failure
											Noninfective gastroenteritis & colitis
											Septic shock
65	1-5yr	F	HP	*P. vivax*	6.2	5.3	5	6	DHA+PIP	High dose	Bacterial meningitis
											Febrile convulsions
											Gastroenteritis and colitis
66	1-5yr	F	LP	Mixed species	.	7.6	6	13	DHA+PIP	High dose	Pulmonary tuberculosis
											Marasmus
											Tinea corporis
67	1-5yr	M	LP	*P. vivax*	11.9	.	.	12	IV artesunate / DHA+PIP	High dose	Tuberculous meningitis
											Septicaemia
											Conjunctivitis

68	>=15yr	M	NP	*P. vivax*	13.2	.	.	7	IV artesunate	Nil	Nontraumatic intracranial haemorrhage
69	>=15yr	M	HP	*P. vivax*	11.2	12.4	4	7	DHA+PIP	High dose	Common cold
											HIV
											Candidiasis
											Tuberculous meningitis
											Acute tubulo-interstitial nephritis
70	5-15yr	M	HP	Mixed species	.	3.3	21	26	DHA+PIP	Low dose	Suspected tuberculosis
71	5-15yr	F	HP	*P. vivax*	11.8	9.4	7	18	DHA+PIP	High dose	Pulmonary tuberculosis
											Constipation
											Acute pharyngitis
											Bacterial meningitis
72	>=15yr	M	HP	Mixed species	.	8.6	23	23	DHA+PIP	Low dose	-
73	>=15yr	F	HP	Mixed species	6.3	.	.	3	IV artesunate / DHA+PIP	High dose	Dehydration
											Lobar pneumonia
											Hypovolaemic shock
74	>=15yr	M	HP	Mixed species	8.2	.	.	9	IV artesunate / DHA+PIP	High dose	Ventral hernia without obstruction or gangrene
											Septic shock
75	>=15yr	M	NP	*P. vivax*	.	.	.	4	IV artesunate / DHA+PIP	Nil	Essential hypertension

Abbreviations: M=male, F=female, HP=Highland Papuan, LP=Lowland Papuan, NP= Non=Papuan, PQ=Primaquine, IV=intravenous, DHA+PIP= dihydroartemisinin-piperaquine
