# Supplementary material for: The risk of adverse clinical outcomes following treatment of Plasmodium vivax malaria with and without primaquine in Papua, Indonesia
Source: PLoS Negl Trop Dis. 2020 Nov 11;14(11):e0008838. doi: 10.1371/journal.pntd.0008838 (PMC7657498; doi:10.1371/journal.pntd.0008838)
Supplement: S6 Table — (PDF) [file pntd.0008838.s008.pdf]

S6 Table: Haematological and clinical details of the 36 patients whose Hb fell by >25% to below 7g/dl

Patient number	Age cat	Sex	Ethnic group	Malaria species at presentation	Hb in g/dl at initial presentation	Lowest Hb within 30 days in g/dl	Outcome at day 30 (death/alive)	PQ dose
1	>=15years	F	LP	Mixed Species	5.8	2.7	Alive	High dose
2	>=15years	M	HP	*P. vivax*	7.8	5.2	Death	High dose
3	>=15years	M	LP	*P. vivax*	4.5	3.2	Death	High dose
4	>=15years	F	HP	Mixed Species	10.1	6.2	Alive	High dose
5	5-14years	F	HP	Mixed Species	4.8	3.6	Alive	High dose
6	1-4years	F	HP	*P. vivax*	13.2	6	Alive	High dose
7	1-4years	F	HP	Mixed Species	6.8	4.1	Death	High dose
8	>=15years	M	HP	Mixed Species	12	4.1	Alive	High dose
9	1-4years	F	HP	Mixed Species	8.8	5.7	Alive	High dose
10	1-4years	F	HP	*P. vivax*	10.7	6.2	Alive	High dose
11	5-14years	F	HP	*P. vivax*	10.5	6.2	Alive	High dose
12	1-4years	M	HP	Mixed Species	8.8	5	Alive	High dose
13	5-14years	F	HP	Mixed Species	9.9	5.6	Alive	High dose
14	5-14years	M	HP	*P. vivax*	9.1	5.7	Alive	High dose
15	1-4years	M	HP	Mixed Species	6.4	4.4	Alive	High dose
16	1-4years	F	HP	*P. vivax*	11.9	4.2	Alive	High dose
17	>=15years	F	HP	Mixed Species	6.9	4.1	Alive	High dose
18	1-4years	F	HP	*P. vivax*	7.2	3	Alive	High dose
19	5-14years	F	HP	*P. vivax*	10.1	6.1	Alive	High dose
20	>=15years	M	HP	Mixed Species	9.6	4.8	Alive	High dose
21	5-14years	F	HP	Mixed Species	8.3	5.4	Alive	High dose
22	5-14years	M	HP	Mixed Species	7.9	5.2	Alive	High dose
23	1-4years	F	HP	Mixed Species	11.2	4.9	Alive	High dose
24	1-4years	F	HP	*P. vivax*	10.8	6.3	Alive	High dose
25	>=15years	F	HP	*P. vivax*	8.1	5.2	Alive	Low dose
26	1-4years	M	NP	*P. vivax*	9.6	6.2	Alive	Low dose
27	1-4years	M	HP	Mixed Species	4	2	Alive	Low dose
28	1-4years	M	HP	*P. vivax*	5.5	2.7	Alive	Low dose
29	>=15years	F	HP	*P. vivax*	8.7	5.4	Alive	Low dose

30	1-4years	F	HP	*P. vivax*	9	5.8	Alive	Nil
31	>=15years	F	NP	*P. vivax*	7.4	5.1	Alive	Nil
32	1-4years	F	HP	*P. vivax*	3.5	2.1	Alive	Nil
33	1-4years	M	HP	*P. vivax*	10.1	3.9	Alive	Nil
34	5-14years	F	HP	Mixed Species	10.1	2.3	Alive	Nil
35	1-4years	F	HP	*P. vivax*	11.4	5.9	Alive	Nil
36	1-4years	F	HP	*P. vivax*	10.2	5	Alive	Nil

Abbreviations: M=male, F=female, HP=Highland Papuan, LP=Lowland Papuan, NP= Non=Papuan, PQ=Primaquine
